# Case report: Abscesses in children caused by invasive group A *Streptococcus*

**DOI:** 10.3389/fmed.2024.1438624

**Published:** 2024-08-09

**Authors:** Danchun Guo, Shuting Zhuang, Qinghua Lu, Yunsheng Chen, Qing Meng, Lifang Sun, Yuejie Zheng, Wenjian Wang, Dingle Yu

**Affiliations:** Shenzhen Children's Hospital, Shantou University Medical College, Shenzhen, Guangdong, China

**Keywords:** group A *Streptococcus*, abscess, child, antibiotics, resistance

## Abstract

*Streptococcus* is one of the common pathogens of suppurative infections. Invasive group A *Streptococcus* (iGAS) infections often develop from skin or soft tissue infections, and streptococcal toxic shock syndrome is considered the main cause of death in Chinese children with iGAS infectious disease. However, soft tissue infections caused by iGAS infections, especially the formation of abscesses, are relatively rare. A retrospective study was conducted, and pediatric in-patients who were diagnosed with an iGAS infection identified by cultures from normally sterile sites and treated in a tertiary hospital during 2016–2018 were included. A total of 14 patients were identified, which included 10 boys and four girls. The patients had an age range from 3 months to 10 years and were diagnosed with soft tissue infections and a formation of abscesses caused by iGAS infections. The most common sites of infections were the lower limbs. In five patients, the abscess was accompanied by fever, and the local soft tissue showed redness, swelling, tenderness, and an elevated skin temperature. Laboratory findings included an increased white blood cell (WBC) count in 12 patients, an increased C reactive protein (CRP) level in seven patients, and an increased erythrocyte sedimentation rate (ESR) in 10 patients. No patients had an elevated procalcitonin level. For all 14 patients, we performed puncture and drainage of abscesses, and cultured GAS from the drainage fluid. All children also received antibiotic treatment. During 2 months of follow-up, the patients' condition remained stable and no evidence of kidney or heart damage was observed. For pediatric patients with abscesses, early diagnosis, prompt treatment with incision and drainage, and immediate culture of the drainage fluid are important. Upon confirmation of an iGAS infection, β-lactam antibiotics should be given to provide effective treatment, and in some patients with poor therapeutic outcomes, the use of vancomycin as an alternative can achieve the desired results.

## Introduction

*Streptococcus pyogenes*, also known as group A *Streptococcus* (GAS), is a pathogen with humans as the main host. GAS is widespread throughout the world and is associated with a wide range of diseases of varying severity, including non-invasive diseases, such as pharyngotonsillitis and impetigo, severe invasive infections, such as septicemia, necrotizing fasciitis, and toxic shock syndrome, and immune-related diseases, such as rheumatic fever and glomerulonephritis (1–3). *Streptococcus* is one of the common pathogens of suppurative infections. Invasive GAS (iGAS) infections often develop from skin or soft tissue infections, and streptococcal toxic shock syndrome (STSS) is the leading cause of death among children with iGAS infectious disease (4). On the one hand, the increase in scarlet fever and iGAS infections in the UK and other European countries in 2022 has caused worldwide concern (2, 3, 5, 6). On the other hand, abscesses caused by iGAS are rarely reported. This retrospective study analyzed the clinical data of children with abscesses caused by iGAS infections in Shenzhen Children's Hospital to explore the clinical characteristics, laboratory results, treatment, and outcomes.

## Case description

### Setting and patients

This was a retrospective study that included 14 patients with GAS cultured from pus punctured from abscesses. From May 2016 to August 2018, 14 patients with iGAS infections were diagnosed through pus culture with a clinical diagnosis of soft tissue infections with abscess formation. The demographics, clinical presentation, treatment, and microbiological data of patients were collected from electronic medical records.

### Bacterial identification and antimicrobial susceptibility testing

Pus samples were inoculated in a 5% defibrillated sheep blood medium, and β-hemolytic colonies were selected for bacitracin detection. Positive strains were identified as GAS by Lancefield group-specific antiserum.

We tested the susceptibility of the 14 strains to eight antimicrobial agents (penicillin, azithromycin, erythromycin, clarithromycin, clindamycin, tetracycline, levofloxacin, and chloramphenicol; Oxoid Limited). Minimum inhibitory concentration (MIC) values were determined according to the guidelines of the Clinical and Laboratory Standards Institute (CLSI; 2016–2018) by using the broth dilution method. Quality control was performed using *Streptococcus pneumoniae* ATCC 49619, which was provided by the Clinical Test Center of the Ministry of Health and maintained by the Microbiology Laboratory of Shenzhen Children's Hospital.

### DNA extraction and *emm* sequence typing

Genomic DNA was obtained from freshly grown GAS culture using a Chelex-based DNA extracting kit for genetic analysis. Following a previously reported protocol (https://www2.cdc.gov/vaccines/biotech/strepblast.asp), *emm* sequence types were determined. The DNA extraction kit, PCR reagents, and primers were all obtained from Shanghai SBS Genetech Co., Ltd. (China). Amplicons were sequenced by Guangzhou BGI Genomics Co. Ltd. The *emm* sequence type was determined based on the sequence identity (>95%) of the first 180 bp of the *emm* gene between the tested sequence and the reference *emm* gene.

### Statement of ethics

This study was approved by the Research Ethics Committee of the Shenzhen Children's Hospital (No. 202107802). Informed consent was obtained from patients or their guardians before sample collection.

## Diagnostic assessment

### Patients' baseline characteristics

A total of 14 hospitalized patients with culture-confirmed abscesses were included. There were 10 male patients (71.43%) and four female patients (28.57%). The age of the patients ranged from 3 months to 10.8 years, and the median age was 1.8 years. No patients had any underlying condition.

### Clinical characteristics

Among the 14 patients, the disease course was 2–18 days, with a median length of 7 days. All 14 patients had inflammatory manifestations, such as localized skin redness, swelling, localized pain, and localized increase in skin temperature. Five patients had fever, while nine patients had no fever. The most common sites of infection were the lower limbs, including the calves in two patients, the knees in two patients, the thighs, hips, feet, and ankles in one patient, the mouth, neck, armpits, hand, elbow plus hand, and the upper limbs in one patient. For all 14 patients, we performed puncture and drainage of abscesses and successfully obtained pus after puncture. After the bacterial culture, all 14 pus samples were confirmed positive for iGAS infection. The details of the specific clinical presentation of the patients are provided in Table 1.

**Table 1:** Clinical data of children with soft tissue infection and abscess formation caused by invasive group A *Streptococcus*.

**No**.	**Gender**	**Age**	**Site of infection**	**Stay days**	**WBC ( × 10^9^/L)**	***N*%**	**CRP (mg/L)**	**ESR (mm/h)**	**PCT (ng/mL)**	**Culture results**	***emm* type**	**Antibiotics**
1	M	3 m	Left foot	2	22.38	60.9	26.5	10	0.92	Pus culture GAS	1.0	AS 2 d + ACP 6 d
2	F	9 m	Right thigh	6	12.97	49.1	8.3	66	0.71	Pus culture GAS	75.0	AS 6 d
3	M	10 m	Left elbow, right hand	18	32.04	64.9	37.3	46	0.73	Pus culture GAS	6.4	AS 18 d
4	M	1 y	Left axilla	3	19.40	-	-	-	-	Pus culture GAS	12.19	CS 3 d
5	M	1 y 5 m	Left region of the neck	5	30.80	64.8	24.3	-	-	Pus culture GAS	12.0	AS 4 d
6	M	1 y 5 m	Right ankle	8	12.35	31.0	< 0.5	41	0.22	Pus culture GAS	2.0	AS 8 d
7	F	1 y 8 m	Floor of mouth	3	20.07	67.5	118.9	-	-	Pus culture GAS	12.66	Cefuroxime 3 d
8	F	1 y 9 m	Right lower leg	12	34.79	80.3	4.5	39	0.01	Pus culture GAS	4.0	Van+CS 11 d
9	M	2 y 9 m	Left hip	3	20.78	63.3	4.1	30	0.08	Pus culture GAS	28.0	Cefuroxime 3 d
10	M	3 y 7 m	Right knee	4	9.09	59.7	15.3	51	-	Pus culture GAS	12.0	CS 4 d
11	M	5 y 10 m	Right index finger	8	21.38	84.6	22.3	57	-	Pus culture GAS	6.4	AS 8 d
12	F	8 y 1 m	Left upper limb	11	26.70	88.3	126	68	0.88	Pus culture GAS	1.9	Cefatriaxone 1 d, Cefatriaxone+Van 6 d, Van 2 d, Cefuroxime 2 d
13	M	9 y	Left lower leg	6	7.99	47.3	2.7	25	-	Pus culture GAS	1.0	AS 6 d
14	M	10 y 8 m	Osteomyelitis of right proximal tibia and osteomyelitis of left knee	14	46.82	89.1	< 0.5	89	< 0.5	Pus culture GAS	12.8	AS 14 d

M, Male; F, Female; y, year; m, month; N, neutrophil; WBC, white blood cell; CRP, C reactive protein; ESR, erythrocyte sedimentation rate; PCT, procalcitonin; GAS, group A Streptococcus; d, day; AS, Amoxicillin and Sulbactam; ACP, amoxicillin clavulanate potassium; CS, cefoperazone and sulbactam; Van, vancomycin.

-, No data or no test.

### Laboratory results

A blood routine initial leukocyte count was quite variable. Leukocytosis was presented in 12 (85.71%) patients (12.35–46.82 × 10^9^/L). C reactive protein (CRP) levels were detected in 13 patients, and seven patients (53.8%, 7/13) had higher levels of CRP (15.3–126 mg/L) than the normal value (10 mg/L). The levels of erythrocyte sedimentation rate (ESR) were detected in 11 patients, and 10 patients (90.9%) had higher ESR levels (25–89 mm/h) than the normal value (15 mm/h). Procalcitonin levels in sera were detected in eight patients, and four patients (50.5%) had higher procalcitonin levels (0.71–0.92 ng/mL) than the normal value (0.5 mg/L). Local ultrasound showed local soft tissue inflammatory thickening/mass with liquefaction. All 14 patients underwent abscess incision and drainage, and the puncture fluid was sent for microbial culture, which indicated the growth of GAS. One patient had a complication of varicella. In this patient, the herpes fluid culture also showed GAS and the throat swab for the GAS antigen was positive. Based on the *emm* typing, the 14 patients showed three strains of *emm*1 and its subtypes, one strain of *emm*2.0 type, one strain of *emm*4.0 type, two strains of *emm*6.4 type, five strains of *emm*12 type and its subtypes, one strain of *emm2*8.0 type, and one strain of *emm*75.0 type. The *emm* typing is summarized in Table 1. All tested strains were phenotypically sensitive to penicillin, levofloxacin, and chloramphenicol. The resistance rate to azithromycin, erythromycin, clarithromycin, clindamycin, and tetracycline was 78.6%. The results of the susceptibility of *S. pyogenes* strains to antimicrobial agents are shown in Table 2.

**Table 2:** Susceptibility of group A *Streptococcus* strains (*N* = 14) to antimicrobial agents.

**Antibiotic**	**R% (*N*)**	**I% (*N*)**	**S% (*N*)**	**(**μ**g/mL)**			
				**Breakpoint**	**MIC50**	**MIC90**	**MIC range**
Penicillin	0	0	100 (14)	S ≤ 0.125	0.004	0.008	0.004–0.008
Azithromycin	78.6 (11)	0	21.4 (3)	S ≤ 0.5 R ≥ 2	>256	>256	0.06–512
Erythromycin	78.6 (11)	0	21.4 (3)	S ≤ 0.25 R ≥ 1	>256	>256	0.125–512
Clarithromycin	78.6 (11)	0	21.4 (3)	S ≤ 0.25 R ≥ 1	>256	>256	0.06–512
Clindamycin	78.6 (11)	0	21.4 (3)	S ≤ 0.25 R ≥ 1	128	256	0.015–512
Tetracycline	78.6 (11)	0	21.4 (3)	S ≤ 2 R ≥ 8	64	64	0.5–64
Levofloxacin	0	0	100 (14)	S ≤ 2 R ≥ 8	1	2	0.25–2
Chloramphenicol	0	0	100 (14)	S ≤ 4 R ≥ 16	2	4	2–4

R, Resistant; I, Intermediate; S, Susceptible; N, number; MIC, Minimum Inhibitory Concentration.

## Details on the therapeutic intervention

The medium hospital stay was 7 days (2–18 days). The details of the therapy for the 14 patients are summarized in Table 1. All 14 patients were treated with intravenous antibiotics. A total of eight patients were treated with amoxicillin sodium sulbactam sodium, two patients with cefoperazone sodium sulbactam sodium, two patients with cefuroxime, one patient with vancomycin + cefoperazone sodium sulbactam sodium, and one patient with ceftriaxone/cefuroxime + vancomycin.

## Follow-up and outcomes

The follow-up for all 14 patients was conducted by telephone. No patients experienced sequelae, such as nephritis, rheumatic fever, and rheumatic heart disease.

## Discussion

GAS can cause a broad spectrum of infections, ranging from minor illnesses, such as pharyngitis and superficial skin infections, to severe and invasive diseases. iGAS infection is a life-threatening condition, with high case fatality and high morbidity rates (7). Surveillance is essential to accurately determine the real burden of this disease in children. According to the Active Bacterial Core surveillance reports of the National Center for Disease Control (CDC), overall iGAS incidence increased every year from 2012 to 2019 (7). iGAS infections are defined as infectious diseases associated with iGAS isolated from a sterile site in the body, such as sepsis, arthritis, pleurisy, and abscesses. All 14 iGAS isolates in this case series were isolated from sterile sites (pus from soft tissue infections), and therefore, all 14 patients were considered to have iGAS infections (7). There is a professional surveillance network for iGAS diseases in North America, while the National Center for Disease Control (CDC) in China only monitors scarlet fever caused by iGAS infection as part of its real-time surveillance of infectious diseases. According to a report from Canada, the incidence of iGAS diseases among aborigines in Alberta increased from 10/100,000 in 2003 to 52.2/100,000 in 2017 (8). In China, GAS-related invasive diseases may be underestimated because they are not routinely monitored.

This case report included more male patients (*n* = 10) than female patients (*n* = 4), which may be related to the greater activity level of boys vs. girls of the same age and thus a greater risk of local injury in boys. In terms of age, half of the patients were in the early childhood period of 1–3 years. During this toddler period, children typically lack a good sense of self-protection and are easily injured, which can lead to localized infections.

*Emm* typing is the most commonly used molecular typing method for the study of GAS. In the present case series, *emm*1 (and its subtypes) and *emm*12 (and its subtypes) accounted for the highest proportion, which is consistent with other reports in China from the past 2 years (9, 10). Other types, such as *emm*2.0, *emm*4.0, *emm*6.4, *emm*28.0, and *emm7*5.0, were detected at lower rates in this case series, indicating that all types of *emm* GAS may cause invasive infections, including the formation of soft tissue abscesses. For example, a study in Finland (11) showed that the *emm* GAS type that causes puerperal infections in young women is *emm*28. An outbreak of invasive GAS disease in New Zealand was confirmed to be caused by an *emm*81 clone (12). A patient with a non-necrotizing soft tissue infection and streptococcal toxic shock syndrome (STSS) caused by a novel *emm* subtype (*emm*76.10) of GAS was reported (13). Other studies found that *emm*1, *emm*28, *emm*42.1, *emm*55, and *emm*25 are more likely to cause invasive infections than other *emm* types (14, 15). Furthermore, the most common *emm* type of GAS varies across different age groups and regions (16–18).

With regard to the pathogens that can easily cause abscesses, the pathogens most commonly reported as the cause of orbital and periorbital abscesses are *Pseudomonas aeruginosa, Streptococcus constellation, Staphylococcus epidermidis, Staphylococcus aureus*, and others (19). However, the 14 children with abscesses in this case series were all infected with iGAS, which has been relatively rarely reported in China.

With regard to the site of infection, among the 14 patients in this series, eight had infections in the lower extremities, distributed among the thighs, knees, legs, and feet. Only three patients had infections in the upper limbs, and the other infection sites were the neck, armpits, and oral cavity. From these cases, it can be seen that abscess formation upon iGAS infection is most likely to occur in the limbs. The reasons behind the limbs being prone to abscesses have been analyzed (20). The hands are the most exposed parts of the body and thus the most vulnerable to injury, allowing them to be easily infected with bacteria. In patients with infections, pus can spread to the deep tissues, permitting abscess formation, which can lead to pain, fever, and other systemic symptoms, including even sepsis. Necrotizing infection is not only a threat to the limbs but also to life. As a result, a hand injury that initially appears to be “trivial” should never be ignored as it can develop into a deep infection that requires drainage, wound debridement, and intravenous antibiotic treatment as soon as it is found. Delayed diagnosis and improper early treatment can quickly lead to the formation of abscesses, the destruction of the normal anatomical structure, and irreparable functional degradation. In addition to abscess formation in the limbs, various reports have shown that iGAS infection can cause iliolumbar abscess (21), abdominal wall abscess (22), lumbar epidural abscess (23), nasal septum abscess (24) spinal epidural abscess (25), brain abscess (26), and so on. Accordingly, abscesses caused by iGAS infections can occur at various sites. Thus, even if the disease is only a superficial soft tissue infection, it should not be ignored and should be treated immediately to prevent aggravation.

Based on the laboratory examination results, leukocytosis was common in the present case series. An increased white blood cell (WBC) count occurred in 12 of the 14 patients, and in nine of the 12 patients, the WBC count was >20 × 10^9^/L. In almost all patients, the WBC count was dominated by an increase in neutrophils, and the proportion of neutrophils was more than 60% in nine patients (9/13). In addition, the level of CRP was generally increased in seven of the 13 patients, among which six patients had CRP levels above 20 mg/L. An increased ESR level was detected in 12 of the 13 patients. However, the procalcitonin level was not increased in the majority of patients, with only four patients (4/14) showing a slight increase (0.71–0.92 ng/mL). The children who were hospitalized for more than 8 days had the following characteristics: a WBC count >20 × 10^9^/L and/or a very high CRP level, exceeding 20 mg/L, as well as an increased ESR level by varying degrees. Therefore, in children with abscesses following soft tissue infections, total leukocyte counts, neutrophil ratios, CRP levels, and blood sedimentation rates can be used as tests that, in combination with essentially normal or slightly elevated calcitoninogen levels, predict local rather than systemic infection. This is consistent with previous findings where it was observed that CRP is more sensitive than procalcitonin in differentiating local bacterial infections (27). Such results need to be validated in future studies.

For the treatment of abscesses, timely determination of the cause of the disease and drainage are necessary. It is crucial to send the drainage fluid for immediate microbiological culture. Antibiotics can be used empirically before the results of the bacterial culture. Then, when the results of the bacterial culture are obtained, the antibiotics can be adjusted according to the results of the antibiotic sensitivity test. According to the Clinical and Laboratory Standards Institute (CLSI) 2020 standard, penicillin and ampicillin are always the first choice for the treatment of hemolytic streptococcal infections. Non-sensitive isolates are extremely rare among any β-hemolytic *streptococcus*, and GAS has not been reported among non-sensitive isolates. All strains analyzed in this study were susceptible to penicillin, levofloxacin, and chloramphenicol. Therefore, penicillin can remain the first-line antibiotic for treating GAS infections in pediatric patients in China. Among the 14 patients, 12 patients were treated with β-lactam antibiotics and two patients were treated with vancomycin. Penicillin and other β-lactam antibiotics are the first choice for the treatment of iGAS infections. Previous studies have mentioned the use of intravenous immunoglobulins (IVIGs) and clindamycin in the treatment of iGAS infections, but these drugs were not used in this series, and despite that, all the patients recovered well (7). Penicillin tolerance has been involved in treatment failures in patients with GAS infections, and some studies have found an association with penicillin tolerance (28, 29).

## Conclusion

GAS is one of the most common pathogens causing abscesses. The WBC count, CRP level, and ESR of patients with this type of abscess usually increase to varying degrees, whereas the procalcitonin level likely remains in the normal range or increases only slightly. After the diagnosis of an abscess is confirmed, the abscess should be drained urgently and the drainage fluid should be sent for microbial culture immediately. β-lactam antimicrobials are the therapeutic agents of choice for the treatment of iGAS infections, and a replacement with vancomycin may be required as an alternative for some patients who do not respond well to treatment.

## Data availability statement

The raw data supporting the conclusions of this article will be made available by the authors, without undue reservation.

## Ethics statement

This study was approved by the Research Ethics Committee of the Shenzhen Children's Hospital (No. 202107802). Informed consent was obtained from patients or their guardians before sample collection. The studies were conducted in accordance with the local legislation and institutional requirements. Written informed consent for participation in this study was provided by the participants' legal guardians/next of kin. Written informed consent was obtained from the individual(s) for the publication of any potentially identifiable images or data included in this article.

## Author contributions

DG: Formal analysis, Methodology, Data curation, Writing – original draft. SZ: Data curation, Formal analysis, Methodology, Writing – original draft, Conceptualization. QL: Data curation, Formal analysis, Writing – review & editing. YC: Writing – review & editing, Resources. QM: Resources, Writing – review & editing. LS: Resources, Writing – review & editing. YZ: Conceptualization, Funding acquisition, Investigation, Supervision, Writing – review & editing. WW: Funding acquisition, Supervision, Writing – review & editing. DY: Conceptualization, Formal analysis, Funding acquisition, Investigation, Methodology, Project administration, Supervision, Writing – original draft, Writing – review & editing.
